# 3D Printing of Biodegradable Polymeric Microneedles for Transdermal Drug Delivery Applications

**DOI:** 10.3390/pharmaceutics16020237

**Published:** 2024-02-06

**Authors:** Faisal Khaled Aldawood, Santosh Kumar Parupelli, Abhay Andar, Salil Desai

**Affiliations:** 1 Department of Mechanical Engineering, College of Engineering, University of Bisha, P.O. Box 001, Bisha 67714, Saudi Arabia; faldawood@ub.edu.sa; 2 Center of Excellence in Product Design and Advanced Manufacturing, North Carolina Agricultural and Technical State University, Greensboro, NC 27411, USA; sparupel@ncat.edu; 3 Champions Oncology, Inc., 1 University Plaza Dr, Hackensack, NJ 07601, USA; dr.abhay.andar@gmail.com

**Keywords:** 3D printing, biomedical, drug delivery, microneedle, polymers, therapeutics

## Abstract

Microneedle (MN) technology is an optimal choice for the delivery of drugs via the transdermal route, with a minimally invasive procedure. MN applications are varied from drug delivery, cosmetics, tissue engineering, vaccine delivery, and disease diagnostics. The MN is a biomedical device that offers many advantages including but not limited to a painless experience, being time-effective, and real-time sensing. This research implements additive manufacturing (AM) technology to fabricate MN arrays for advanced therapeutic applications. Stereolithography (SLA) was used to fabricate six MN designs with three aspect ratios. The MN array included conical-shaped 100 needles (10 × 10 needle) in each array. The microneedles were characterized using optical and scanning electron microscopy to evaluate the dimensional accuracy. Further, mechanical and insertion tests were performed to analyze the mechanical strength and skin penetration capabilities of the polymeric MN. MNs with higher aspect ratios had higher deformation characteristics suitable for penetration to deeper levels beyond the stratum corneum. MNs with both 0.3 mm and 0.4 mm base diameters displayed consistent force–displacement behavior during a skin-equivalent penetration test. This research establishes guidelines for fabricating polymeric MN for high-accuracy and low-cost 3D printing.

## 1. Introduction

Microneedle (MN) technology consists of an array of needles in a micrometer size ranging up to 2 mm in height [1]. MNs deliver active pharmaceutical agents or drugs into the human body by piercing micrometer holes into the skin with minimally invasive action [2,3,4,5]. MN technology provides several benefits compared to traditional patches and syringe injections, which include a painless experience [6], ease of use [7], a reduced chance of infection [8], and capacity for self-administration [9]. Moreover, it also enables the delivery of a wide range of therapeutics into the skin layers with minimally invasive pain [10]. MNs are generally categorized into solid MNs, coated MNs, hollow MNs, dissolvable MNs, and hydrogel MNs [11,12,13,14,15]. Applications of MNs include transdermal drug delivery [16,17], vaccine delivery [18,19,20], cosmetic [21,22], disease diagnostics [23], tissue engineering [24,25], and therapeutic applications. With the increase in the demand for healthcare applications and services, the market size of medical devices was about $600 billion in 2022 and is estimated to reach above $950 billion by 2031 [26]. The market share of MNs also has a significant impact on the healthcare and medical industries as it has applications in a wide range of areas [4] [27,28,29]. According to a recent report, the market size of global microneedle drug delivery systems was about $2.65 billion in 2021 and about $2.83 billion in the following year [30]. The healthcare market research reports that the market size of microneedles will grow dramatically with a compound annual growth rate (CAGR) of 17.8% and is estimated to reach about $7.8 billion by 2027 [31].

Initially, the concept of MNs was proposed for the first time in 1998 on silicon wafers by applying ion etching and photolithography techniques for transdermal drug delivery application [32]. Different manufacturing methods [33,34] such as deep reactive ion etching, laser ablation, extruding polymer molds, laser machining [35], photolithography, and dry etching have been used to manufacture microneedle arrays for different applications. With the advancement in manufacturing techniques [36,37,38,39], materials [40], and pre-/post-processing methods [41], there has been a significant innovation in the MN field. Over the past decade, extensive research studies have been conducted on the fabrication of microneedles using various materials such as glass, ceramic, metal, and polymers [42,43,44,45]. Many of these traditional manufacturing methods have limitations such as being complex and labor-intensive, multiple manufacturing steps, limited design flexibility, and lack of cost-efficiency. Thus, there is a need to develop robust, cost-effective techniques for the fabrication of MNs [46,47,48]. Additive manufacturing (AM), popularly known as 3D printing, is a technique that builds three-dimensional objects in a layer-by-layer manner from the bottom up using a digital computer-aided design (CAD) file [49,50,51,52]. The unique advantages of AM techniques such as a high degree of design flexibility, a wide range of materials, a single-step process, and cost-effectiveness make it an ideal choice for the fabrication of MN arrays and molds. Moreover, the advantages of using AM technology to manufacture MN over traditional methods include fast customization [53], the flexibility of design parameters [54], and the real-time sensing of biological analytics [55].

Extensive research was conducted by various industries and academia to enhance the fabricated microneedle quality, and repeatability using AM processes [56,57,58,59,60]. Caudill et al. demonstrated the AM technique as a rapid, complex, cost-effective, and large-volume manufacturing process to fabricate customized microneedle master molds. In 2019, Johnson et al. reported the fabrication of a microneedle master mold using a commercial 3D printer [54]. In the same year, Krieger et al. introduced a new method called the “print and fill” method for fabricating microneedle molds using a low-cost desktop SLA 3D printer [61]. Moreover, Mathew et al. utilized the digital light processing (DLP) 3D-printing method to manufacture the hollow MN array for transdermal drug delivery application [62]. Various additive manufacturing techniques that have been demonstrated in the literature for manufacturing MNs include selective laser melting (SLM) [63], fused deposition modeling (FDM), and continuous liquid interface production (CLIP) [64]. The printing accuracy and mechanical strength of the MN array have a significant impact on the MNs to accomplish effective transdermal drug delivery. However, these aforementioned studies applied multiple and long pre-processing techniques to enhance the accuracy of the printed microneedles. Additionally, there are very limited research studies reported on the effect of MN design parameters such as the array size, width, aspect ratio, and tip radius on the fabricated MN arrays. The detailed parametric analysis of MNs is of paramount importance as they have a major impact on the MN array’s transdermal drug delivery administration. Current methods to fabricate microneedles are labor-intensive and expensive. Thus, simplifying the manufacturing process to meet the design specifications of therapeutic microneedles is a missing gap in the industry.

In this study, we demonstrate a low-cost 3D-printing process to fabricate a high-quality biodegradable polymer MN array with superior mechanical strength. The biodegradable polymer MN arrays were fabricated using the stereolithography (SLA) technique, with different aspect ratios including 2:1, 3:1, and 4:1. Initially, the 3D-printed mold was fabricated, and then the female MN molds were developed using polydimethylsiloxane (PDMS) material. Further, the biodegradable polymer MN array was fabricated by using the PDMS female molds. The printing accuracy of the MN array and the biodegradable MN array was determined using an optical microscope and scanning electron microscopy. A fracture test under axial force was performed to evaluate the mechanical behavior of biodegradable polymeric MN using the Instron Material Testing System. Additionally, the insertion studies on the parafilm sheet (mimicking artificial skin) were investigated to pierce the MNs into the skin. This study provides a cost-effective manufacturing method to fabricate biodegradable MN array patches for advanced therapeutic and drug delivery applications.

## 2. Materials and Methods

The stereolithography (SLA) technique was implemented for fabricating high-quality microneedle arrays with 50 µm feature resolution using biocompatible and biodegradable materials. Due to the efficiency and safety of the printed MN, it is considered an excellent candidate for transdermal drug delivery applications. The proposed method allows us tp have superior control over the introduced parameters such as height, tip sharpness, and aspect ratio. MNs with different aspect ratios were fabricated, and analysis of their mechanical strength and piercing was conducted.

### 2.1. Experimental Procedures

**Figure 1:**
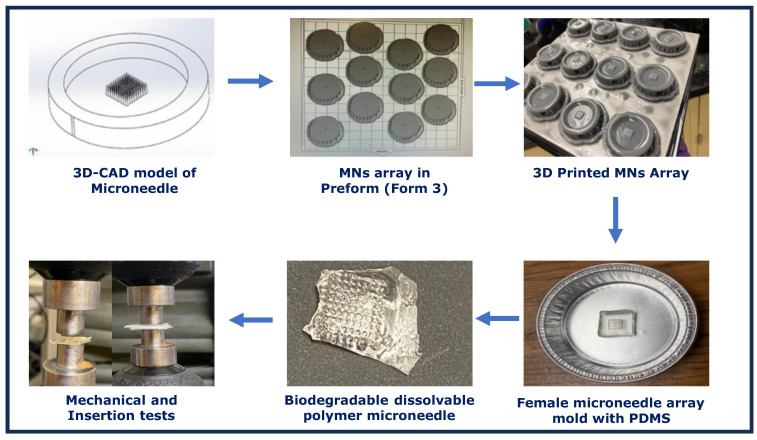
Experimental procedures of microneedle array using additive manufacturing method.

Figure 1 illustrates the steps for the fabrication of biodegradable MN arrays using AM method. Initially, the 3D CAD model of the MN arrays was designed using SolidWorks software version 2022. The 3D model was further sliced and exported in standard triangle language (.STL) format. The 3D-printed MNs were peeled from the build platform and rinsed in the ethanol solution for 10 min to remove the unwanted residue. The 3D-printed MNs were placed in an LC-3D print box UV post-curing unit at 60 °C for 20 min to attain smooth surface finish. Further, the PDMS female mold was developed from these 3D-printed MNs, which was further casted using the Gantrez™ material. The printing accuracy of the 3D-printed MN and the biodegradable MNs was determined using the optical microscope (Olympus SC50) and scanning electron microscope (SEM) (Zeiss Auriga Crossbeam FIB-FESEM). The mechanical strength and insertion test analysis was conducted to ensure the efficiency of the biodegradable microneedle for further therapeutic applications.

### 2.2. Design

In this study, six conical MN arrays designs—five height values (0.6, 0.8, 0.9, 1.2, and 1.6) and three aspect ratios (2:1, 3:1, and 4:1)—are investigated. Each array has a total of 100 needles per patch (10 × 10). The impact of different aspect ratios on the MNs was investigated.

### 2.3. Material

Tough 1500 photopolymer resin material was used to 3D-print the MN arrays in this research. This material supports printing resolutions of 50 and 100 microns in the layer thickness [65]. Moreover, this material is resilient, biocompatible, tough, and durable. In addition, Tough 1500 Resin is certified to be safe for skin contact, which makes it an ideal material for fabricating biomedical devices. The anhydrous ethanol obtained from Fisher Chemical was used to cure the 3D-printed MN array. Dow Sylgard™ 184 Silicone Elastomer, a polydimethylsiloxane (PDMS), was obtained from Ellsworth Adhesives, Germantown, WI, USA. PDMS material was used to develop the female MN array mold. Gantrez™ AN-169, alternating copolymers of methyl vinyl ether (MVE) and maleic anhydride, was sourced from Ashland. Poly (ethylene glycol) with average Mn20,000 and Caffeine powder were obtained from Sigma Aldrich, Burlington, MA, USA. The combination of Gantrez™ and Caffeine powder (X *w*/*v* %) was used to fabricate the biodegradable MN array patch. Parafilm M^®^. (Sigma Aldrich), a flexible thermoplastic sheet with 0.13 mm thickness, was used as a skin simulator (artificial skin) for insertion studies.

### 2.4. Drug-Loading Preparation

**Figure 2:**
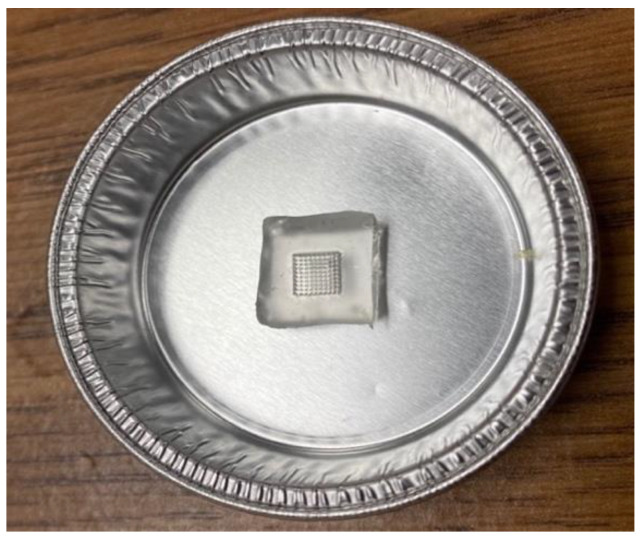
PDMS female microneedle array mold.

The PDMS used in this study (Dow SYLGARDTM 184 Silicone Encapsulant Clear) was prepared by mixing polymer resin and a curing agent in 10:1 weight ratio. PDMS material was then casted into the microneedle arrays and placed in an oven at 45 °C overnight to develop the female MN array mold. The female PDMS mold was further placed in an aluminum dish (Figure 2). The biodegradable polymer MN was prepared by mixing the drug (caffeine) (5%*w*/*w*), polymer—Gantrez™ (20%*w*/*w*), and PEG (2%*w*/*w*) materials with the icy-cold distilled water at a speed of 1000 rpm overnight. The following day, this polymer mixture was placed on a hot plate and heated at 80 °C, and then was poured in the female PDMS MN mold. Additionally, the entire mold was placed inside a vacuum-tight desiccator for 10 min, in order to get rid of air bubbles and allow the polymer mixture to conform well to the mold.

### 2.5. Mechanical Testing

**Figure 3:**
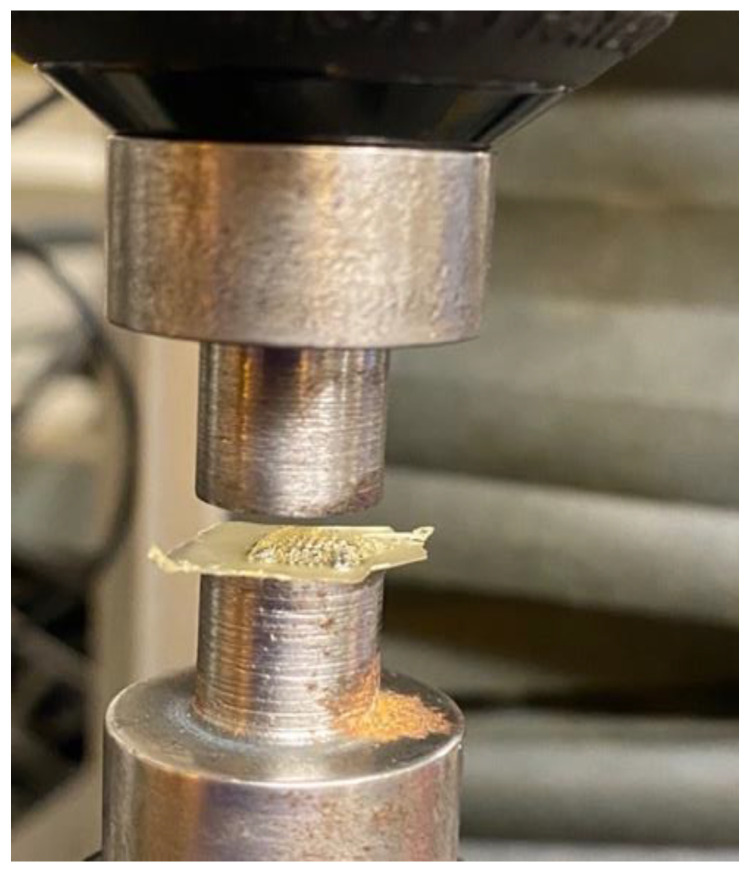
Mechanical station for fracture test under axial force.

A fracture test under axial force was performed to evaluate the mechanical behavior of polymeric MN array, to ensure that the microneedle will not bend or fracture during the insertion into the skin. This test was conducted using an Instron Material Testing 5542A System machine station. Initially, the polymeric microneedles were attached to the fixture as shown in Figure 3 and were pressed against a flat metal block attached to the moving head station. The maximum force of 50 N was applied in the y-axis direction with a speed of 0.5 mm/min. The continuous force and displacement data of the MN array was recorded at regular time intervals of 100 ms per frame to determine the point of MN failure. This analysis was designed to determine the mechanical strength of the polymeric MN array patch needed to pierce the skin for drug delivery applications.

### 2.6. Insertion Test

The effects of the biodegradable polymeric MN array on the force required to pierce the skin were investigated using parafilm sheets. Parafilm sheets have been used in previous studies to perform an MN insertion test to mimic the human skin [49]. Arshad et al. conducted a study for the assessment of microneedle performance using parafilm films for in vitro insertion [66]. The study concluded that 92% of the loaded drug was released within about 2 h, which allowed effective drug dosage delivery. Moreover, Larrañeta et al. argued that using parafilm is suitable for initiating quality check of the fabricated MN and also serve as a test to compare the insertion efficiency of several prospective MN formulations [67]. Moreover, Liu et al. conducted an insertion test for a polymer MN into the parafilm and porcine skin, and concluded that the mechanical property of MN was analogous in both models for reliable testing [68]. Another study proposed a facial system to study the permeation performance of drug by dissolving MN. The MN were placed into dissolution bath; the molecules of the drug were released successfully [69]. Thus, based on proven background and literature studies, our team has chosen parafilm as a first-stage skin model, which will be augmented with porcine and more sophisticated models during in vitro testing in our next phase of research.

**Figure 4:**
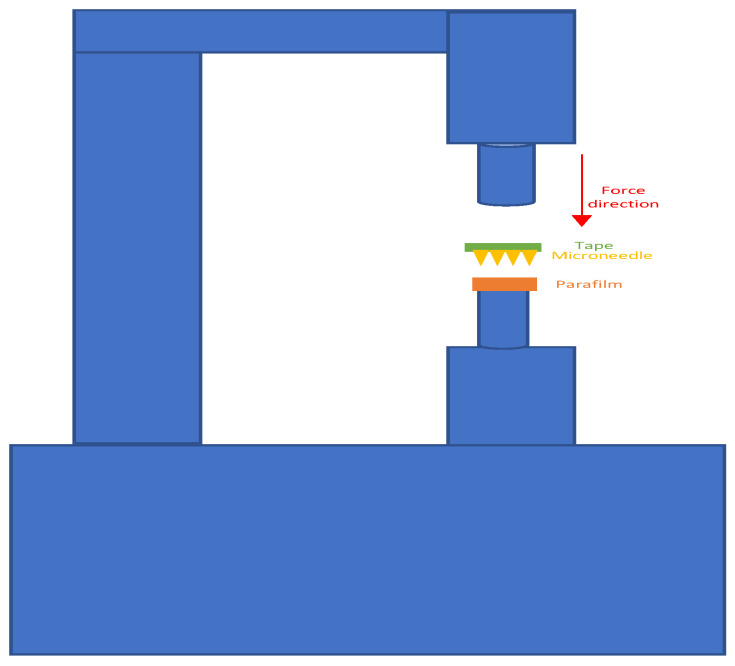
Microneedle insertion test setup.

An Instron Material Testing 5542 System was utilized to conduct the insertion test analysis. The polymeric MN was glued to a circular microscope cover glass to ensure equal distribution of the force on the microneedle surface with 12 mm diameter and 0.16 mm thickness. Then, the polymeric MN array was taped and placed above the Parafilm sheets. Prior to testing, the Parafilm (at 0.13 mm thickness) was folded into multiple layers to be used as model to mimic the human skin in an insertion test. The maximum force applied in this test was set at 50N with a speed of 5 mm/s. The workstation setup for the insertion test is shown in Figure 4. To evaluate the depth of the insertion test, we calculate the number of parafilm sheets that have been penetrated using microneedle, then multiply by 0.13 mm, which is the thickness of one parafilm sheet. Moreover, continuous force and displacement measurements were recorded to determine the point of needle insertion.

## 3. Results

**Figure 5:**
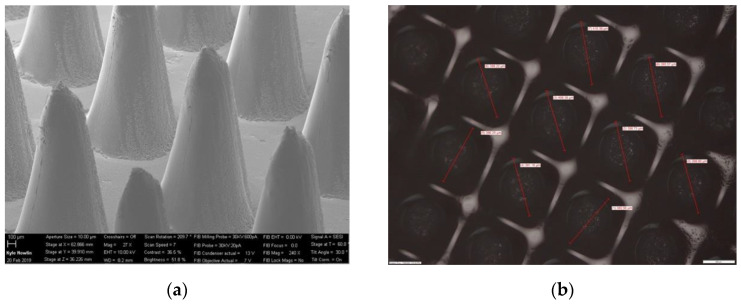
(**a**) SEM image of 3D-printed MN array—side view, and (**b**) optical microscope image of MN array—top view.

**Figure 6:**
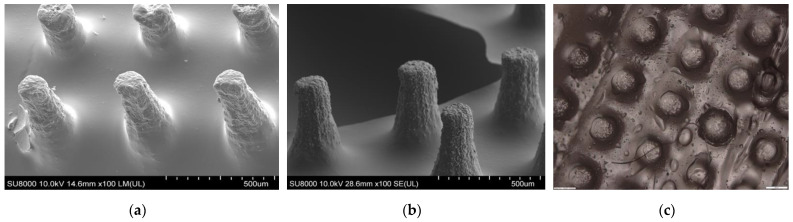
(**a**,**b**) SEM image of polymeric MN array—side view, and (**c**) optical microscope image of polymeric MN array—top view.

A biodegradable polymeric MN was fabricated by manufacturing an MN array with a commercial 3D printer (Formlabs Form 3). Moreover, the printing time of one microneedle array is estimated to be around 20 min. Caffeine powder was successfully loaded, and polymeric microneedles were produced for further analysis. The research investigated the printing accuracy of the height and diameter of the MN array (Figure 5) and polymeric MN (Figure 6) using an optical microscope and SEM, respectively. Finally, the polymeric MN was tested for mechanical properties by applying axial force and insertion tests.

### 3.1. Printing Accuracy of 3D-Printed and Biodegradable Polymer MN Array

The printing accuracy was evaluated in terms of the diameter and height of the MN array using an optical microscope and SEM, respectively. The height of the printed MN array values were shorter for all the MN arrays compared to the original CAD model. Table 1 and Table 2 represent the measurement values for the printed MN’s diameter and height using a scanning electron microscope. For the 0.30 mm-diameter MN array, the values of the MN tip height ranged from 0.52 mm to 0.58 mm for 0.6 mm, 0.80 mm to 0.85 mm for 0.90 mm, and 1.10 mm to 1.16 mm for 1.20 mm, respectively. On the other hand, for the 0.40 mm-diameter MN array, the values of the MN tip height ranged from 0.70 mm to 0.77 mm for 0.80 mm, 1.10 mm to 1.15 mm for 1.20 mm, and 1.51 mm to 1.56 mm for 1.60 mm, respectively. Similarly, the difference between the printed microneedles was between 92% and 97% for the 0.3-diameter and 92% and 94% for the 0.4-diameter MNs, respectively. Overall, the printed diameters were highly consistent with the CAD model as compared to the heights. This can be attributed to the viscous flow of the biodegradable Gantrez material leading to difficulty in filling the deeper and narrower regions of the microneedle mold. The tip diameters of the polymer microneedles after the casting process varied from 40 to 80 µm. These tip diameters are depicted in Figure 6 and were further post-processed in an acetone vapor chamber [70] to smoothen the microneedle profiles and sharpen the tip diameters. The post-processed microneedle tip diameters varied from 18 to 48 µm depending on their aspect ratio and base diameter dimensions.

The difference between the 3D-printed and biodegradable polymer MN array’s height and diameter was less than 10% and 5%, respectively, compared to the CAD model. In comparison to other studies [54,71], these differences show a significant improvement in the literature wherein researchers employed different pre-processing techniques. Johnson et al. used open-source software to slice the CAD files of MN at a 0.001 mm slice thickness and these leads improved the intended dimensions of the fabricated MN [72]. Moreover, Kundu et al. argued that an anti-aliasing process is required to improve the dimensional accuracy of a conical design of the MN [73].

### 3.2. Mechanical Testing of Biodegradable Polymeric MN

**Figure 7:**
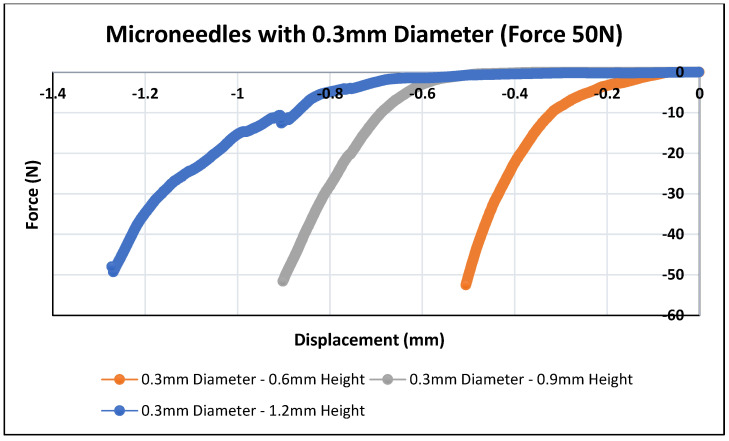
Polymeric MN mechanical testing (MNs of 0.3 mm in diameter, n = 5).

**Figure 8:**
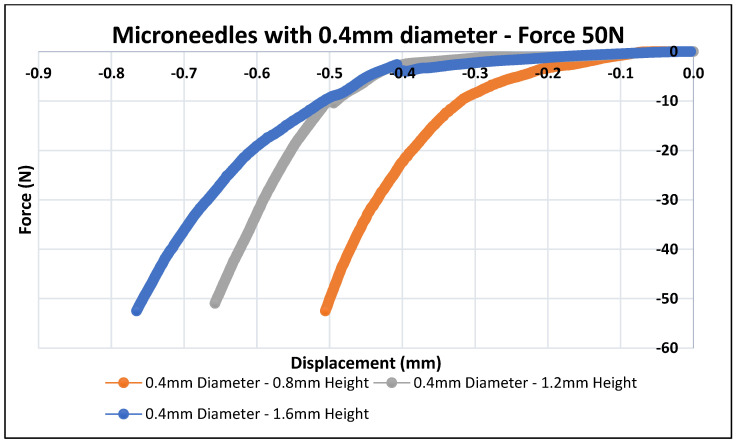
Polymeric MN mechanical testing (MNs of 0.4 mm in diameter, n = 5).

Skin is an elastic part of the body, which possesses significant sturdiness. Therefore, the MNs must be capable enough to penetrate the skin and stratum corneum layer without any fracture or breakage of needles during skin insertion [63]. In this study, the polymeric MN arrays were exposed to forces of up to 50 N to determine the displacement depths of the MN arrays using the texture analyzer set up as shown in Figure 3. The mechanical performance of the biodegradable polymeric MN array was evaluated by applying an axial force to ensure that the MNs were mechanically robust enough to withstand the minimum insertion force required to penetrate the skin. The force–displacement curves as illustrated in Figure 7 and Figure 8 were plotted for the different-aspect-ratio MNs with their respective diameters to analyze the mechanical performance. Table 3 illustrates the displacement of microneedle array dimensions under axial force at 50 N. 

Figure 7 and Figure 8 show force–displacement plots for MNs with different aspect ratios and diameters to provide guidance on the strength and penetration capabilities. The MN displayed varying levels of force–displacement behavior based on their aspect ratios and base diameters. All the polymer microneedles tested underwent an elastic deformation perpendicular to the axial force, eventually transitioning to plastic deformation and failure at the maximum load of 50 N. The total load of 50 N was selected as that is far in excess to the practically feasible load to be applied to microneedles during their application as patches on the human dermal surface. The polymeric MN array with a 0.3 mm diameter had a larger displacement compared to the 0.4 mm-diameter one. This can be attributed to the larger volume for the 0.4-mm-diameter MNs, resulting in a higher stiffness during axial compression. MNs with higher aspect ratios (Design 2 and 3) and a 0.3 mm base diameter had a higher deformation as compared to Design 1 (2:1 aspect ratio). Design 4 (0.4 mm diameter and 2:1 aspect ratio) has the least displacement (0.5 mm) as compared to higher-aspect-ratio needles (Design 5 and 6). Different needle designs were considered as the stratum corneum penetration force can be dependent on the location and type of skin membrane on the human body. Thus, taller and thicker needles would be needed (Designs 4–6) to penetrate robust skin surfaces such as the one on the sole of feet as compared to the delicate skin on the arm. MNs with a 0.4 mm base diameter offered a higher resistance during deformation and stalled at lower displacement as compared to the 0.3 mm-diameter MNs. However, these needles were able to provide a higher strength for penetration and would be applicable for the thicker skin penetration regions of the body. Overall, all the MN designs performed their intended penetration function beyond baseline compression force levels (10 N) expected of MNs for skin penetration. Shah et al. reported in their study the fabrication of an MN array with a solvent-casting process using biodegradable polymer material [74]. In their study, all the fabricated MN arrays exhibited a fracture force greater than the minimum force required to penetrate the skin for an MN array, which is 4 N. Ning et al. reported that the minimum force required to penetrate the single MN was 0.058 N per needle or 5.8 N per array of 100 needles [75]. Chi et al.’s research group reported the fabrication of three types of dissolvable MNs using hyaluronic acid (HA) with various molecular weights (10K, 74k, and 290k). At a 500 μm displacement, the compression forces were 21.6, 13.9, and 5.9 N per array of 100 microneedles, for 10k-HA-MN, 74k-HA-MN, and 290k-HA-MN, respectively [76]. As compared to the prior literature, it can be inferred that all the fabricated MN arrays in our research study are mechanically robust enough to withstand the minimum force required to pierce the human skin.

### 3.3. Insertion Test of Polymeric MN

**Figure 9:**
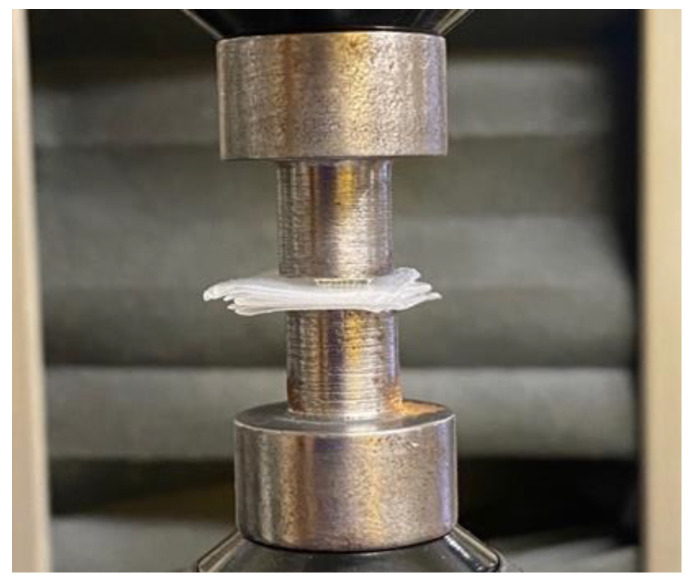
Insertion test setup.

Biodegradable polymer MN arrays fabricated using the 3D-printed mold were tested for their skin penetration capability by utilizing a texture analyzer. It is essential for the MN to possess sufficient mechanical strength to be pierced into the skin successfully without any failure during the insertion through the skin. Larraneta et al. reported that, even though the parafilm sheets present moderately lesser penetration depths compared to the porcine skin, it could still be utilized as a promising material to supersede biological tissue for piercing studies [67]. The average thickness of a single parafilm sheet is 127 ± 3.560 µm. The depth of the MN array penetration into the parafilm was determined using an Instron Material Testing 5542 System. The piercing test was conducted (Figure 9) effectively with no MN failure or damage. The displacement measurements of each aspect ratio’s MN array was measured. During this insertion analysis, the MN array and parafilm sheet would distort once they come into contact with each other. Further, with a gradual increase in the pressure applied, the MN array plunged through the parafilm sheets without any damage to the MNs. Table 4 illustrates the depth of penetration of different designs of the MN array including aspect ratios of 2:1, 3:1, and 4:1.

**Figure 10:**
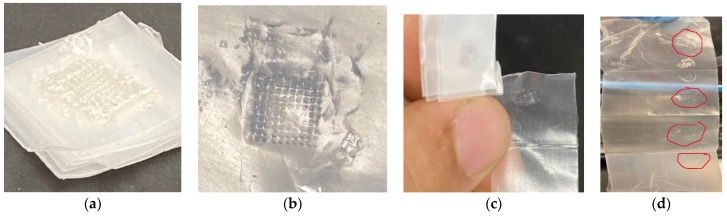
Parafilm after insertion test: (**a**) side view; (**b**) top view; (**c**) individual parafilm; and (**d**) unfolded parafilm sheets.

Figure 10 illustrates the parafilm after the insertion test using biodegradable polymer microneedles. As can be seen from Figure 10c,d, multiple layers of the parafilm were penetrated by the microneedle array depending on their height. The MN array with a higher aspect ratio (4:1) penetrated deeper into the parafilm sheets compared to the lower-aspect-ratio (3:1 and 2:1) MN arrays. The depth of penetration for the 4:1-aspect-ratio-design MNs was 1.17 mm and 1.43 for an MN height of 1.20 mm and 1.60 mm, respectively. In this case, it shows clearly that the needle with a thicker diameter (0.4 mm) penetrated with a lower depth percentage (89%) compared to the needle with a thinner diameter (0.3 mm) (97.5%), whereas the depth of penetration for the 2:1-aspect-ratio-design MNs was 0.52 mm and 0.65 for an MN height of 0.60 mm and 0.80 mm respectively. Thus, from the insertion analysis, it can be inferred that the biodegradable MN array fabricated in this research study can penetrate depths ranging between 0.52 mm and 1.60 mm.

As can be seen in Table 3, the tip diameters for higher-aspect-ratio needles were smaller as compared to lower-aspect-ratio needles. Further, larger-base-diameter MNs had larger tip diameters. It is important to note that smaller tip diameters aid in the easy insertion of MNs within the skin, thereby requiring lower penetration forces [77]. However, too-fine or small-tip diameters can result in the premature fracture of the MNs during insertion [78]. Thus, an optimal tip diameter is desirable based on both the base diameter and aspect ratio of the MNs. In our research, the tip diameters varied between 36 to 18 µm for the 0.3 mm-base-diameter MNs and 45 to 24 µm for the 0.4 mm-base-diameter MNs, respectively. These tip diameters were effective in the uniform penetration of the parafilm films (skin-equivalent model) as shown in Figure 7 and Figure 8.

The percentage difference between the microneedle height and the depth of the needle penetrated through the parafilm sheets was less than 19% for all the MN array designs, this percentage is acceptable, as shown in a previous study [75]. The results of the analysis demonstrate that the MNs were adequately resilient and had the potential to penetrate the parafilm sheets. The typical thickness of the stratum, corneum, and epidermis is between 0.01, 0.02 mm, and 0.1 mm, respectively. The results shown in Table 3 emphasize that the biodegradable polymeric MN array is mechanically strong enough to penetrate actual human skin and can be utilized for transdermal drug delivery applications. Accordingly, the results prove that biodegradable polymeric MN arrays successfully pierce through the stratum corneum and could reach the dermis layer of the human skin for drug release applications.

### 3.4. Comparative Analysis between AM and Laser Ablation Method

In this section, a comparative analysis of the fabricated MN array using the AM method and laser ablation method was evaluated as shown in Table 5. For the fabrication time of a single MN array, it takes approximately between 5 to 10 min for an MN array with 100 milliseconds to approach the burn point for a single microneedle in the desired polymer sheet [79]. On the other hand, AM takes between 20 to 30 min depending on the number of needles in the array [80]. However, the AM method enables the printing of MNs with complex geometry and a customized design [54], wherein the laser ablation method is limited to conical profiles with restricted aspect ratios. Moreover, laser ablation cannot scale up for large-scale production [81]. Even though the AM method offers customized profiles, it requires a high-quality 3D printer [9] to reach higher resolutions [82]. On the other hand, the laser ablation method produces MNs with fine accuracy [83]. In addition, AM requires post-processing steps before having an array ready to use, such as removing support material and curing [84]. In contrast, laser ablation does not require any further steps. In order to prepare female MN molds for therapeutic applications, the laser ablation method required double-casting [85], compared to single-casting when applying the AM method [86].

## 4. Limitations and Future Work

Even though the geometry of the 3D-printed MN arrays were acceptable and slightly different compared to the original CAD model, further post-processing techniques can be utilized to enhance the current designs. Thus, one could manipulate different parameters of the designed MN array such as the layer thickness, material type, printing resolution, and manufacturing method. In this study, our group successfully fabricated a biodegradable polymeric MN. However, further investigation is required, such as with high-performance liquid chromatography (HPLC) to determine the drug release content and an in vivo study to validate the fabricated biodegradable polymeric MN. In addition, a finite element analysis test could possibly validate the current results of the mechanical test for the printed MN array. Finally, in vitro cytotoxicity, biocompatibility, and degradation tests are proposed in our next phase of research to understand the stability and drug elution properties of our microneedles.

## 5. Conclusions

In this research study, we demonstrated the fabrication of high-quality 3D-printed MN arrays using stereolithography (SLA) AM techniques for transdermal drug delivery application. SLA technology was utilized to 3D-print the MN array, followed by a PDMS female MN mold to fabricate the biodegradable polymeric MN. The study proposed six designs consisting of different diameters and aspect ratios with a total of 100 MN for each array. The study investigated the effects of the aspect ratio and diameter on the biodegradable MN array. The dimensional accuracy and mechanical behavior of the polymeric MN were investigated using optical microscope, SEM, mechanical, and insertion tests, respectively. With the difference of the printed MN array height of 10% and diameter of 5% relative to the original CAD design, the proposed method illustrates the capability of producing a high-quality MN array. The mechanical tests concluded that the height of the needle and the aspect ratio have a significant effect on the mechanical behavior of polymeric MN. The insertion tests clearly indicated the validity of the MNs to penetrate through the stratum corneum to the dermis layers for drug delivery applications. Finally, this study demonstrated the fabrication of biodegradable polymeric MN arrays with superior print quality and robust mechanical strength. This research establishes a framework for the design and manufacturing of biodegradable MN arrays for therapeutic applications.

## Figures and Tables

**Table 1:** Dimensions of printed 0.3 mm diameter MN array using optical microscope and SEM.

Diameter = 0.3 mm (n = 16)				
	Aspect Ratio	2:1	3:1	4:1
**Design MN**	height	0.60	0.90	1.20
**Diameter of Printed MN (mm)**	max	0.312	0.309	0.329
	min	0.283	0.291	0.293
**Printed to Design** **(Diameter)**	% difference from mean	97.00%	95.00%	92.19%
**Height of Printed MN (mm)**	max	0.58	0.85	1.16
	min	0.52	0.80	1.10
**Printed to Design** **(Height)**	% difference from mean	96.67%	94.44%	86.67%

**Table 2:** Dimensions of printed 0.4 mm diameter MN array using optical microscope and SEM.

Diameter = 0.4 mm (n = 16)				
	Aspect Ratio	2:1	3:1	4:1
**Design MN**	height (mm)	0.80	1.20	1.60
**Diameter of Printed MN (mm)**	max	0.439	0.416	0.397
	min	0.362	0.382	0.363
**Printed to Design** **(Diameter)**	% difference from mean	94.25%	95.50%	92.75%
**Height of Printed MN (mm)**	max	0.77	1.15	1.56
	min	0.70	1.10	1.51
**Printed to Design** **(Height)**	% difference from mean	97.50%	95.83%	87.50%

**Table 3:** Displacement of biodegradable MN array under axial force at 50 N.

Design #	Diameter (mm)	Aspect Ratio	Height (mm)	Tip Diameter (µm)	Displacement (mm) at Force = 50 N
**1**	0.3	2:1	0.6	36	0.51
**2**	0.3	3:1	0.9	25	0.89
**3**	0.3	4:1	1.2	18	1.20
**4**	0.4	2:1	0.8	45	0.50
**5**	0.4	3:1	1.2	37	0.65
**6**	0.4	4:1	1.6	24	0.76

**Table 4:** Depth of penetration of the biodegradable MN array.

Design #	Diameter (mm)	Aspect Ratio	Height (mm)	Depth of Penetration (mm)	Percentage of Penetration Depth to Needle Height (%)
**1**	0.3	2:1	0.6	0.52	87
**2**	0.3	3:1	0.9	0.78	87
**3**	0.3	4:1	1.2	1.17	97.5
**4**	0.4	2:1	0.8	0.65	81
**5**	0.4	3:1	1.2	1.04	87
**6**	0.4	4:1	1.6	1.43	89

**Table 5:** Comparative analysis between laser ablation method and additive manufacturing method.

Aspect to Compare	Laser Ablation	AM	Reference
**Fabrication time**	Faster	Longer	[79,80]
**MN design**	Not suitable for large-scale production	Complex geometry and customized design	[54,81]
**MN resolution**	Fair accuracy	High accuracy	[82,83]
**Post-processing**	No further steps	Remove support materials and curing	[84]
**Prepare female MN mold**	Double-casting	Single-casting	[85,86]

## Data Availability

The data presented in this study are available upon request from the corresponding author.
